# Supplementary material for: Does AlphaFold2 model proteins’ intracellular conformations? An experimental test using cross-linking mass spectrometry of endogenous ciliary proteins
Source: Commun Biol. 2023 Apr 15;6:421. doi: 10.1038/s42003-023-04773-7 (PMC10105775; doi:10.1038/s42003-023-04773-7)
Supplement: Supplementary file 2 — Supplementary Figures [file 42003_2023_4773_MOESM2_ESM.pdf]

Supplemental Figures

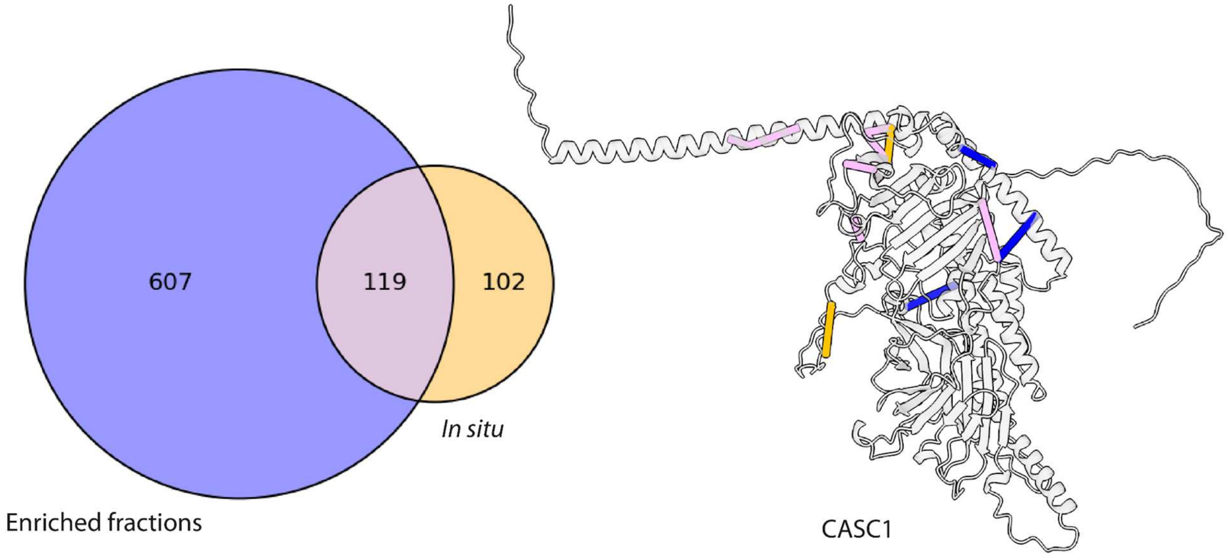

Figure S1. Overlap between cross-linking performed *in situ* and on native protein extracts. The Venn diagram shows counts of unique cross-links observed within the subset of proteins found in both the enriched and *in situ* cross-link sets. (Cross-links are provided on the supporting Zenodo page.) We highlight the CASC1 protein to show the cross-links that were obtained exclusively from the enriched fractions and from the *in situ* dataset as well as the cross-links that were observed in both sets.

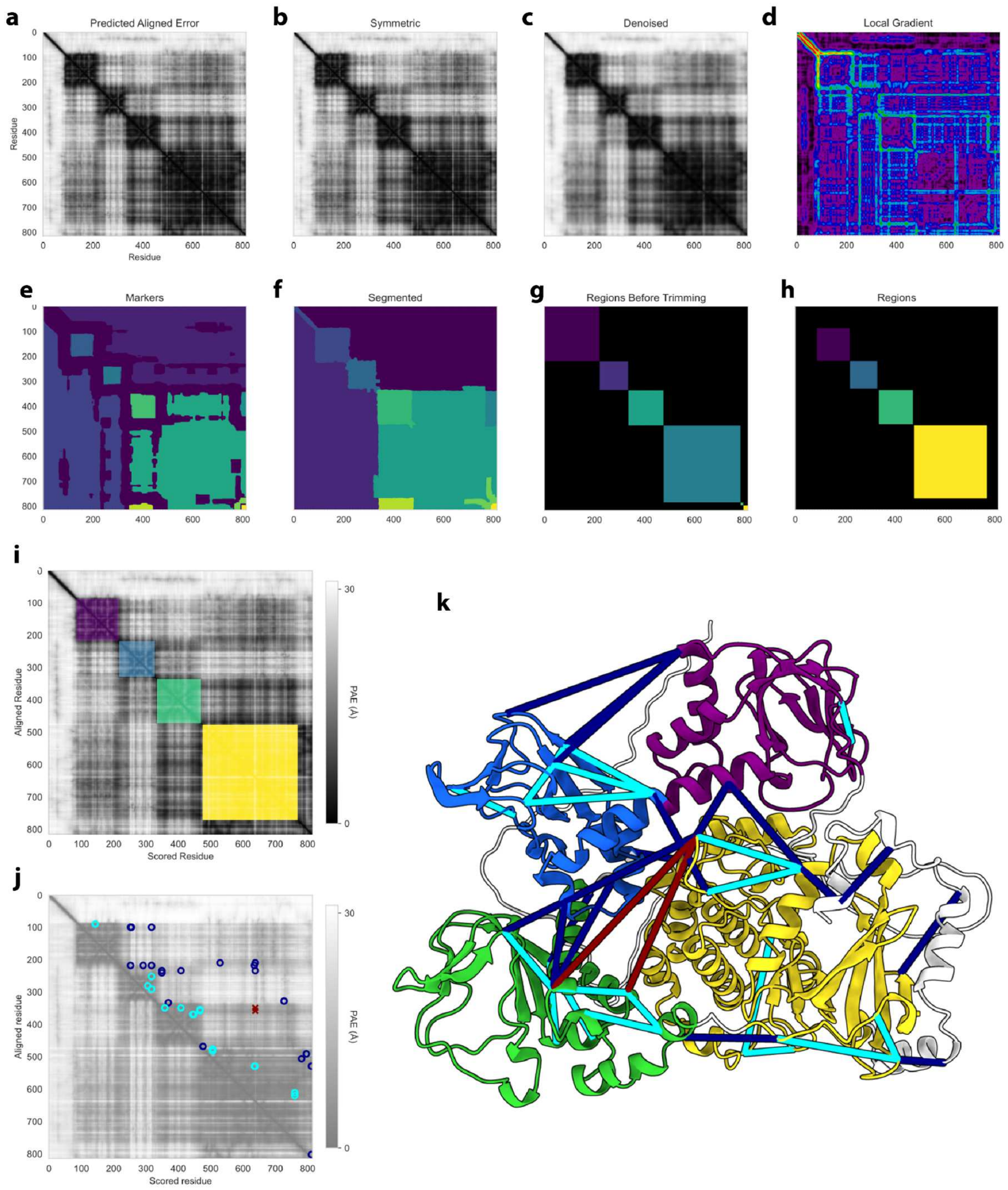

Figure S2. An example of cross-link violations between well-folded regions as determined by watershed segmentation of the PAE score matrix for the AlphaFold-predicted structure of protein PRKG1. Panels show the following different stages in the PAE segmentation algorithm: **a)** Original matrix of predicted aligned error (PAE) for the AF2 structure of the *T. thermophila* protein PRKG1 (Uniprot accession W7XAA6), as predicted using pTM model 1 with relaxation. **b)** PAE matrix made symmetric by averaging with its transpose. **c)** Symmetric PAE matrix after denoising by a median filter. **d)** Local gradient filter applied to the

matrix from C. **e)** Threshold filter is applied to a local gradient matrix in order to identify low-gradient areas as initial basins for watershed algorithm. **f)** Labels for each matrix position determined by watershed algorithm from scikit-image, flooding from the initial basin markers based on the local gradient. **g)** Region labels were extracted for each residue by marching along the diagonal of the labels in matrix F. These region labels are visualized in a matrix where the label value is filled where the x-axis and y-axis residues had the same region label. **h)** Matrix visualization of final region labels where the segmentation labels from F met a width threshold. **i)** Well-folded regions determined by watershed segmentation overlaid on the PAE. **j)** The PAE matrix for PRKG1 with satisfied and violated cross-links plotted onto the matrix. Dark blue circles are the satisfied cross-links inter-region, cyan are satisfied cross-links intra-region, and red x's are the cross-link violations inter-region. **k)** Predicted 3D structure of PRKG1 colored by watershed segmentation region label, with cross-links shown as lines, showing high concordance between the experimental XL/MS data and the AlphFold-predicted model in the folded-domains and restriction of disagreements between domains. Dark blue cross-links are satisfied and inter-region, cyan are satisfied and intra-region, and red are violated and inter-region. Code and data supporting this figure are available on the supporting Zenodo repository.

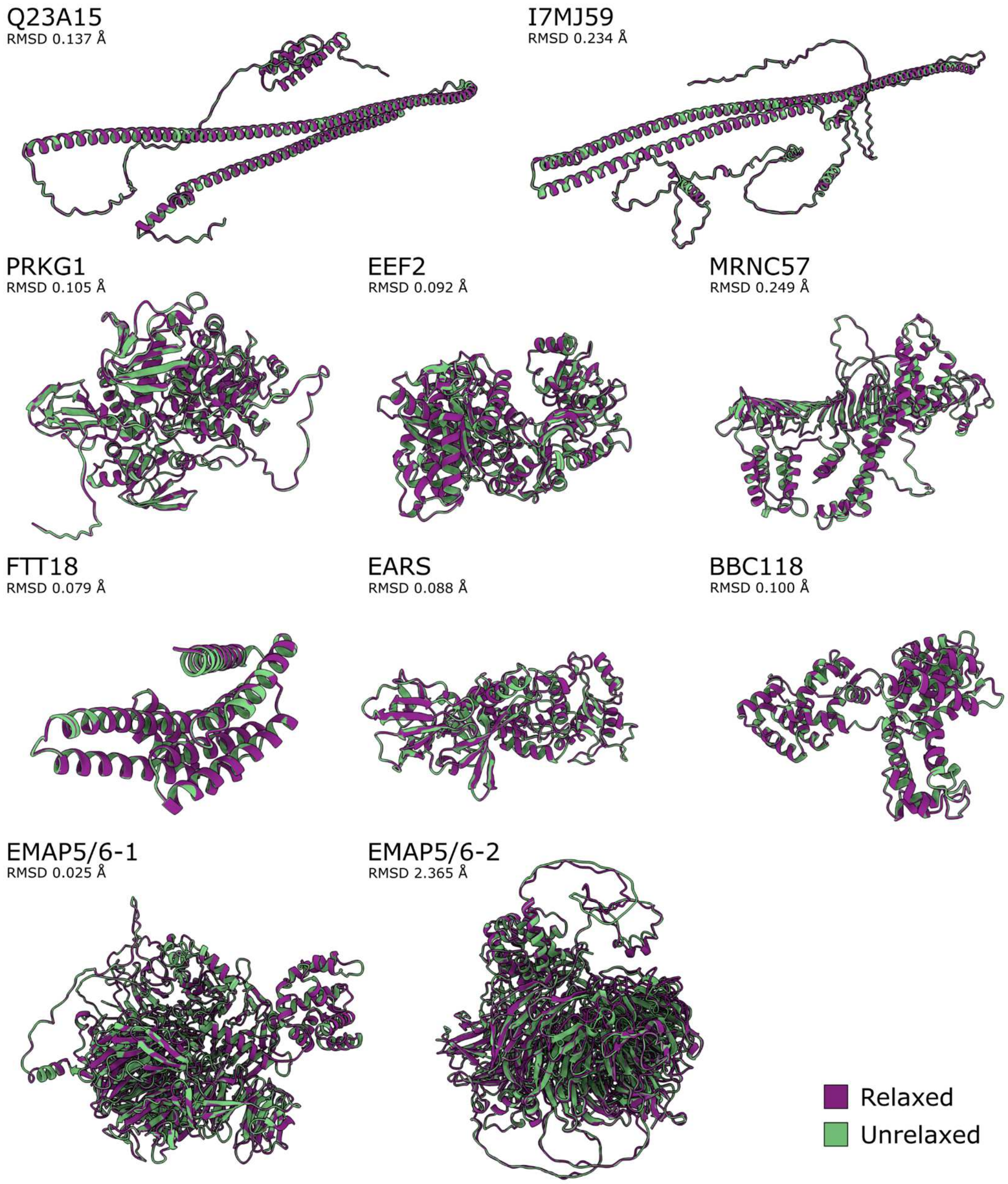

Figure S3. Relaxed and unrelaxed AlphaFold2 structure predictions for the top 10 most cross-linked proteins broadly agree, with a median RMSD of 0.103. The structures for the 10 *T. thermophila* proteins with the most cross-links were predicted by AF2 with and without relaxation. We used the ChimeraX matchmaker command to align the alpha carbons of the unrelaxed structure to the relaxed structure and calculated the root-mean-square deviation between the aligned atoms.

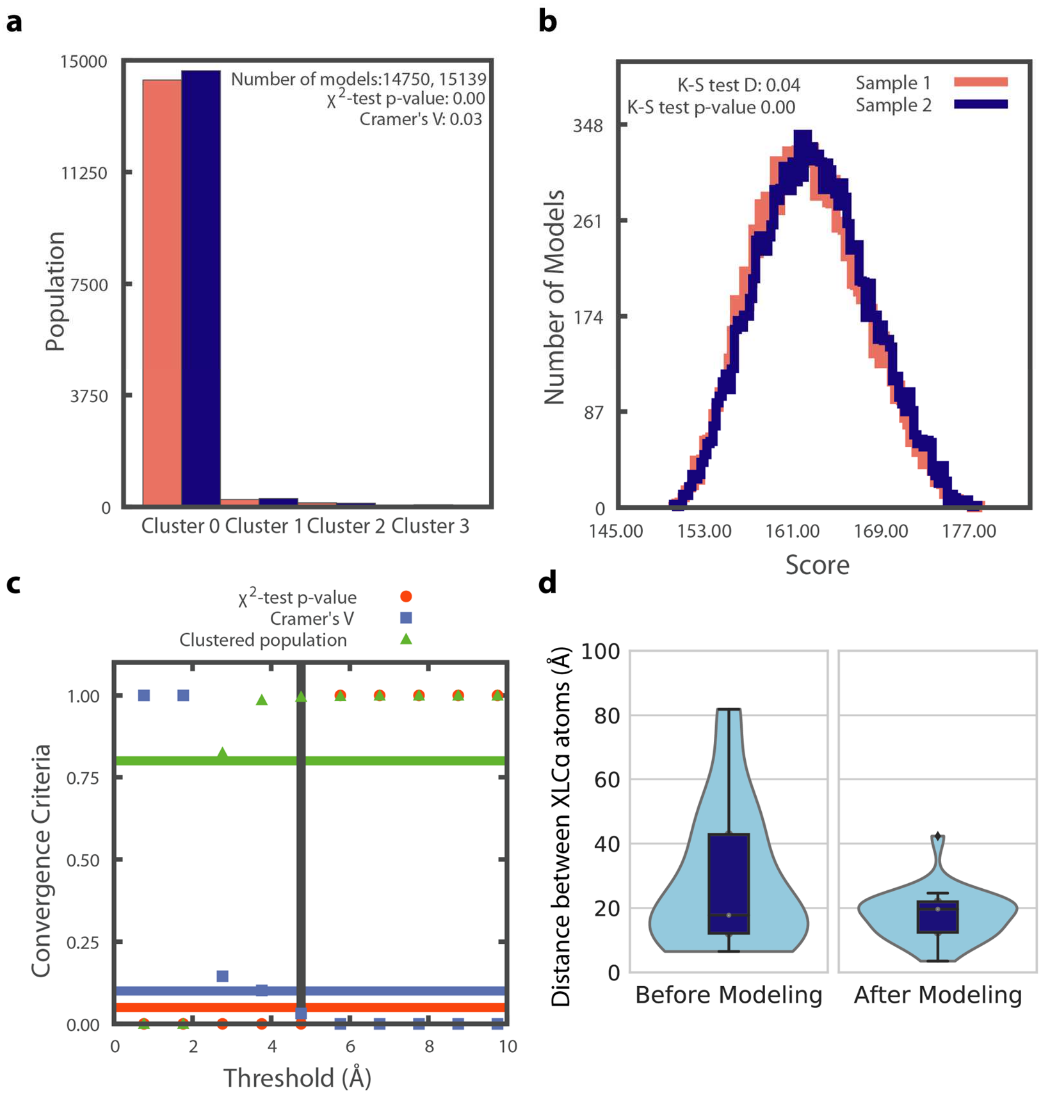

Figure S4. Sampling exhaustiveness and improvement of the BBC118 model using the DSSO cross-links. a) Integrative modeling of BBC118 produced a total of ~30,000 high-scoring models. 29,000 models make up the largest cluster with a cluster precision of 3.369 Å. **b)** Randomly splitting the models into 2 samples and assessing the samples to determine if they come from the same parent distribution confirms that the models' score distributions are similar (i.e., exhibit a small two-sample K-S test D), indicating that the models had converged. **c)** The sampling precision is 4.760 Å as defined and explained in (55). **d)** The distribution of cross-link distances before and after modeling indicate that the modeled conformation of BBC118 could satisfy nearly all of the cross-links. Box plots denote median, first, and third quartiles, and whiskers denote minimum/maximum. (Raw data for the ~30K BBC118 models are available on the supporting Zenodo repository.)

Protein	XL distance (Å) in monomer	XL distance (Å) in oligomer
DYH3	236.6	28.2
DYH4	224.4	24.2
DYH4	228.0	26.9

Table S1. The largest cross-link (XL) violations in the *T. thermophila* outer dynein arm dynein heavy chain structures are satisfied by considering the oligomeric structure. Three cross-link pairs from our data showed large (> 200 Å) violations when compared to the known monomeric ODA protein structures (PDB: 7MOQ). ODA structures were fit into the subtomogram average (accession EMD-9023) of an ODA polymer, and cross-link distances were calculated as intermolecular interactions between copies of the same protein as they exist on microtubules, showing a decrease in distance between cross-linked Cα-atoms to below the expected 30 Å maximum length.

				Fraction Agreement		
**Protein**	**Uniprot**	**Average pLDDT**	**Number of XLs**	**30 Å Threshold**	**35 Å Threshold**	**40 Å Threshold**
PRKG1	W7XAA6	78.04	38	0.95	1.00	1.00
EEF2	Q22DR0	89.48	35	0.71	0.80	0.86
MRNC57	Q236M7	68.39	34	0.74	0.74	0.79
I7MJ59	I7MJ59	67.92	30	0.50	0.50	0.60
EMAP5/6-1	I7LUL6	76.30	30	1.00	1.00	1.00
BBC118	I7ME23	85.48	25	0.60	0.64	0.72
FTT18	I7M2E3	95.14	25	0.84	0.84	0.84
EARS	I7MD81	91.72	24	1.00	1.00	1.00
EMAP5/6-2	I7LU47	75.77	22	1.00	1.00	1.00
Q23A15	Q23A15	75.53	20	0.60	0.75	0.80
KARS	I7LT60	84.99	18	0.67	0.67	0.78
PRKAR1A	Q240X5	76.86	18	1.00	1.00	1.00
HSPA4	I7M4E9	85.20	17	1.00	1.00	1.00
CAPN-1	I7M2X8	75.30	17	0.82	0.94	0.94
I7MKN3	I7MKN3	72.77	17	0.94	1.00	1.00
SARS	Q22Z34	86.57	17	1.00	1.00	1.00
I7M6H8	I7M6H8	81.03	16	0.56	0.63	0.81
SPAG17	Q231F7	67.80	15	0.93	0.93	0.93
I7M3K6	I7M3K6	77.85	15	1.00	1.00	1.00
GNPDA1/2	Q246C5	89.78	15	0.80	0.93	0.93
PGM1	Q22UZ5	94.20	14	1.00	1.00	1.00
BBC73	I7M0S7	86.69	14	0.79	0.79	0.79
IDP2¬†	Q23W20	95.63	14	0.79	0.86	0.86
UBXN2A/B	Q22GI2	52.97	14	0.79	0.79	0.86
CASC1	I7LV93	83.40	13	1.00	1.00	1.00
jacalin-1	Q23JX3	72.12	13	0.85	0.85	0.85
Q234E6	Q234E6	79.71	13	0.92	1.00	1.00
I7M328	I7M328	89.78	13	1.00	1.00	1.00
I7LWT9	I7LWT9	97.17	13	0.85	0.85	0.92
ACO1-2	I7MEK2	94.46	13	0.92	1.00	1.00
AK1-1	I7M2R5	81.29	13	1.00	1.00	1.00
IGR3	Q244Y1	83.06	12	0.83	0.92	0.92
TRAF3IP1	Q24FQ8	66.89	12	0.58	0.58	0.75
Q23F83	Q23F83	96.77	12	1.00	1.00	1.00
Q23DV1	Q23DV1	73.44	12	0.75	0.92	1.00

TTC18	I7MN06	80.92	12	0.92	1.00	1.00
Q24C62	Q24C62	75.14	11	1.00	1.00	1.00
I7M2E8	I7M2E8	95.93	11	1.00	1.00	1.00
I7M350	I7M350	93.94	11	0.73	0.82	0.91
Q23TY1	Q23TY1	94.30	11	0.82	0.82	0.91
Q22AS9	Q22AS9	87.02	11	0.91	1.00	1.00
ME1/2/3	Q24I35	88.12	11	0.82	0.91	0.91
IFT172	Q236H1	82.79	11	0.91	0.91	0.91
CCDC96	I7M6D6	74.58	11	1.00	1.00	1.00
HSP90AA1	Q22W82	85.64	11	1.00	1.00	1.00
I7LXF9	I7LXF9	92.50	11	0.91	1.00	1.00
I7MHP2	I7MHP2	79.25	11	1.00	1.00	1.00
FBPA	I7M007	96.46	11	0.91	1.00	1.00
TRS1	Q23K70	70.66	10	0.70	0.80	0.80
Q22T19	Q22T19	63.39	10	1.00	1.00	1.00
LRS1	I7LXS6	90.45	10	1.00	1.00	1.00
EIF4A	Q23U16	86.97	10	0.60	0.80	1.00
PGI1	Q22B85	96.60	10	0.80	0.80	0.80
PGK1	Q24CJ7	95.65	10	1.00	1.00	1.00
I7MDK2	I7MDK2	92.74	10	1.00	1.00	1.00
I7LUZ1	I7LUZ1	94.29	9	1.00	1.00	1.00
DPY30-1	Q233B2	89.09	9	1.00	1.00	1.00
ACBD7	Q237T8	87.30	9	1.00	1.00	1.00
I7MFS4	I7MFS4	72.76	9	1.00	1.00	1.00
RACK1	Q24D42	86.83	9	1.00	1.00	1.00
VMA5	Q234G6	89.61	9	1.00	1.00	1.00
Q24FH6	Q24FH6	94.73	9	1.00	1.00	1.00
SPEF2	Q22AX1	64.37	9	0.56	0.56	0.67
I7M2A9	I7M2A9	82.61	9	1.00	1.00	1.00
RPS0	I7ME40	86.75	9	0.56	0.56	0.67
ILS1	I7M3D3	90.56	9	1.00	1.00	1.00
DIC3	Q23FU1	81.04	9	1.00	1.00	1.00
Q24CJ0	Q24CJ0	95.49	9	1.00	1.00	1.00
BBC52	I7M8Z8	86.07	9	0.67	0.78	0.89
RANGAD1	I7M486	88.95	9	1.00	1.00	1.00
I7MCM4	I7MCM4	73.10	9	1.00	1.00	1.00
MRNO36	I7MJ14	84.93	9	1.00	1.00	1.00
Q22MP6	Q22MP6	59.57	9	0.44	0.44	0.67

Q24GN6	Q24GN6	74.41	9	0.78	0.78	0.89
DARS	I7M3W6	90.22	8	0.88	0.88	0.88
I7M0R3	I7M0R3	72.38	8	0.75	0.88	1.00
EPC1	Q23FB7	68.05	8	0.75	0.88	1.00
YARS	I7M9D6	91.56	8	0.88	1.00	1.00
DIC2	I7M008	78.09	8	0.75	1.00	1.00
I7ML23	I7ML23	91.83	8	0.88	0.88	0.88
I7LX35	I7LX35	85.79	8	1.00	1.00	1.00
MPK3	I7MKQ5	84.30	8	0.75	0.88	0.88
VMA1	I7MK09	90.48	8	0.88	1.00	1.00
CDC27	I7M395	62.77	8	0.88	1.00	1.00
NPEPL1/LAP3	I7MH91	90.18	8	1.00	1.00	1.00
DLD	Q23DV6	95.02	8	0.75	0.88	0.88
GRS1	Q234B0	91.38	8	0.88	0.88	0.88
EF1A	Q22GX4	89.20	8	0.88	1.00	1.00
I7LW80	I7LW80	68.54	8	1.00	1.00	1.00
TFA	I7MMZ2	91.97	8	0.75	0.75	0.75
I7LZE3	I7LZE3	85.90	7	1.00	1.00	1.00
I7M4K0	I7M4K0	68.90	7	1.00	1.00	1.00
DRH29	Q24DC9	82.16	7	0.86	0.86	1.00
PRS1	I7M6I2	88.67	7	1.00	1.00	1.00
Q23FF1	Q23FF1	63.03	7	0.57	0.86	0.86
CCDC81-1	I7M688	68.91	7	0.86	0.86	0.86
I7MEJ5	I7MEJ5	80.42	7	1.00	1.00	1.00
I7MHD4	I7MHD4	95.69	7	1.00	1.00	1.00
I7LTB9	I7LTB9	38.95	7	0.57	0.57	0.57
I7LZI8	I7LZI8	97.07	6	0.83	0.83	0.83

Table S2. Cross-link agreement with AF2 structure predictions. Cross-link statistics are summarized on a per protein basis.
